# Prevalence of asymptomatic hyperuricemia and its association with prediabetes, dyslipidemia and subclinical inflammation markers among young healthy adults in Qatar

**DOI:** 10.1186/s12902-022-00937-4

**Published:** 2022-01-14

**Authors:** Yasemin Al Shanableh, Yehia Y. Hussein, Abdul Haseeb Saidwali, Maryam Al-Mohannadi, Budoor Aljalham, Hamnah Nurulhoque, Fahad Robelah, Areej Al-mansoori, Susu M. Zughaier

**Affiliations:** 1 College of Medicine, QU Health, Qatar University, PO Box 2713, Doha, Qatar; 2 Biomedical and Pharmaceutical Research Unit, QU Health, Qatar University, PO Box 2713, Doha, Qatar; 3 College of Medicine, Qatar University, PO Box 2713, Doha, Qatar

**Keywords:** Uric acid, Asymptomatic hyperuricemia, Subclinical inflammation, Prediabetes, Qatar Biobank

## Abstract

**Aim:**

The aim of this study is to investigate the prevalence of asymptomatic hyperuricemia in Qatar and to examine its association with changes in markers of dyslipidemia, prediabetes and subclinical inflammation.

**Methods:**

A cross-sectional study of young adult participants aged 18 - 40 years old devoid of comorbidities collected between 2012 and 2017. Exposure was defined as uric acid level, and outcomes were defined as levels of different blood markers. De-identified data were collected from Qatar Biobank. T-tests, correlation tests and multiple linear regression were all used to investigate the effects of hyperuricemia on blood markers. Statistical analyses were conducted using STATA 16.

**Results:**

The prevalence of asymptomatic hyperuricemia is 21.2% among young adults in Qatar. Differences between hyperuricemic and normouricemic groups were observed using multiple linear regression analysis and found to be statistically and clinically significant after adjusting for age, gender, BMI, smoking and exercise. Significant associations were found between uric acid level and HDL-c *p* = 0.019 (correlation coefficient -0.07 (95% CI [-0.14, -0.01]); c-peptide *p* = 0.018 (correlation coefficient 0.38 (95% CI [0.06, 0.69]) and monocyte to HDL ratio (MHR) *p* = 0.026 (correlation coefficient 0.47 (95% CI [0.06, 0.89]).

**Conclusions:**

Asymptomatic hyperuricemia is prevalent among young adults and associated with markers of prediabetes, dyslipidemia, and subclinical inflammation.

## Introduction

Hyperuricemia is a pathophysiological condition observed in association with chronic inflammatory diseases such as rheumatoid arthritis, diabetes, and cardiovascular and kidney diseases [1, 2]. Hyperuricemia results from the overproduction of uric acid, the end product of purine catabolism. Accumulation of uric acid is due to increased production of uric acid in the body coupled with the paucity of the degradative enzyme, uricase [3]. Humans lack the uricase enzyme, which converts uric acid to a more soluble and excretable product, allantoin [4]. Uric acid is released from the cells in the soluble form; however, after exceeding 404 µmol/L (6.8 mg/dL) concentration in a solution, monosodium urate crystals (MSU) begin to precipitate, especially in the settings of other potentiating factors such as low temperatures and acidic environments [5–7]. Uric acid has antioxidant properties when present in body fluids at normal physiological levels. However, uric acid has paradoxical effect and appears to be pro-inflammatory at higher concentrations [8]. Uric acid is mostly excreted through the kidneys by tubular secretion and through the intestines (to a lesser extent); however, elevated uric acid is implicated in kidney stone formation [9].

Asymptomatic hyperuricemia, hyperuricemia in individuals who have not experienced gout nor nephrolithiasis, is viewed as a non-pathological condition whilst its prevalence is estimated to range between 10 and 20% worldwide [10]. Until recently, it was thought that uric acid in the soluble form is inert and does not initiate an inflammatory response. Traditionally, the long-established sole consequences of hyperuricemia have been increased susceptibility to gout and renal nephrolithiasis, and thus international evidence-based guidelines recommend hyperuricemia treatment to target gout and nephrolithiasis only [11–14]. However, Braga et al. have shown that soluble uric acid can activate the NLRP3 inflammasome through the production of mitochondrial reactive oxygen species [15]. Inflammasome activation in immune cells leads to the release of proinflammatory cytokine IL-1β [16]. Recent studies demonstrated that soluble uric acid modulates autophagy and inflammasome activation during bacterial infections [17]. Therefore, some have suggested that soluble uric acid is a danger associated molecule (DAMP) [18]. However, Mendelian randomization studies, which could detect pertinent evidence of a causal relationship, generally failed to find a causal relationship between serum uric acid levels and chronic inflammatory diseases [19–21].

Although many studies investigated the association between uric acid levels and the incidence of chronic inflammatory diseases, few papers have investigated the association between uric acid and hematological indicators in healthy individuals. Insight into the relationship between serum uric acid and subclinical changes in the levels of hematological indicators may yield valuable information about the association between asymptomatic hyperuricemia and chronic inflammatory diseases. The objective of this study is to assess whether asymptomatic hyperuricemia may lead to preclinical, subtle changes in the levels of blood markers in apparently healthy individuals. Such knowledge may shed light on the presence of subclinical metabolic disturbances that precede the incidence of clinically apparent disease.

## Methods

### Study design

This is a cross-sectional study of young adult participants, aged 18-40 years old and devoid of comorbidities. Data were collected from Qatar Biobank [22] on 871 de-identified participants after obtaining ethical approval from QU-IRB and QBB-IRB (RES-ACC-0193-0116). Qatar Biobank cohort data used in this study were collected between 2012 and 2017. The cohort exclusion criteria were: co-morbidities, chronic diseases such as cardiac or renal diseases, leukemia or any neoplasm, type 2 diabetes mellitus, and use of anti-uricosurics drugs, which raise serum uric acid and lower urine uric acid, such as diuretics and salicylates.

All measurements of variables were collected using Qatar Biobank established protocol [23]. Briefly, participants’ blood levels of uric acid, Triglycerides, LDL, HDL, glucose, c-peptide, HbA1c and ferritin were measured at Hamad Medical Corporation (HMC) laboratories, Department of Laboratory Medicine and Pathology (DLMP) that holds College of American Pathologist (CAP) accreditation. Uric acid and lipids profiles were measured using the micro-volume automated biochemical analyser. Glucose was measured by enzymatic method with amperometric detection (Nova statstrip and Roche Accucheck Inform II devices). C-peptide was measured using ECLIA Sandwich technique and HbA1c was measured using latex agglutination inhibition with spectrophotometry (Siemens DCA vantage device). Complete blood count and WBC differential was also performed at the same time point for each participant at the same laboratories using micro-cuvette technology using HemoCue WBC DIFF analyser. Monocytes percentage was provided as part of the CBC and differential count. The subclinical inflammation biomarker Monocyte percentage to HDL ratio (MHR) was calculated by dividing monocyte percentage on HDL-c concentration. Waist circumference was measured slightly below or around the umbilicus, and hip circumference was measured at the widest part of the hip. Seca non-stretchable sprung measuring tape was used and measurements were recorded in centimetres (cm).

### Statistical analysis

All analyses were computed using Stata statistical software package 16 (STATA 16), (Stata Corp, College Station, TX, USA). The prevalence of asymptomatic hyperuricemia in our cohort was investigated. In this study, we defined hyperuricemia as a uric acid level of 356 µmol/L (6.0 mg/dL) or higher, as recommended by a recent paper that explored the suitability of different thresholds [9].

Initial analysis involved drawing histograms and Q-Q plots to examine the distribution of all continuous variables. Qualitative assessment by 3 investigators was carried-out to determine whether variables showed normal or non-normal distribution. Non-normally distributed variables were noted. The predictor variable was serum uric acid. The outcome variables were hematological indices (Hemoglobin, RBC count, TIBC, Folate, White blood cell, Monocyte Percentage (%)); lipid profile variables (HDL-c, LDL-c, Triglycerides); glycemic markers (Random Glucose, C-peptide, HbA_1c)_; and subclinical inflammation markers (Ferritin, and monocyte to HDL ratio (MHR)).

The baseline characteristics of the cohort were summarized into Table 1. For normally distributed variables, the means and standard deviations were presented, but for non-normally distributed variables, the medians and interquartile ranges were presented instead. The associations between serum uric acid levels and the outcome variables were investigated using t-tests followed by correlation tests and multiple linear regression after adjusting for confounders. Statistical significance was determined at α < 0.05.

The cohort was divided based on uric acid levels into a normouricemic and a hyperuricemic group. The means and standard deviations (or medians and interquartile ranges, for non-normally distributed variables) of the outcome variables in the two groups were calculated and the p-values were produced using t-tests for normally distributed variables and Mann-Whitney U tests for non-normally distributed variables.

Two-way scatter plots and corresponding residual vs. fit (RVF) plots, investigating the relationships between uric acid and the outcome variables, were drawn for all variables, to qualitatively determine whether the relationships between uric acid levels and the outcome variables satisfied the linear assumptions required for Pearson’s correlation test. Outcome variables that had a relationship with uric acid that did not satisfy these assumptions were noted. Moreover, outliers in the outcome variables were determined if they were more than 3 interquartile ranges above or below the upper quartile or lower quartile of their distribution, respectively, before investigating the correlation; significant outliers were removed. Pearson’s correlation coefficient (r) and coefficient of determination (R^2^) comparing serum uric acid to outcome variables were calculated. For outcome variables that did not satisfy the required linear assumptions, Spearman’s rank correlation coefficient (ρ) was presented.

Furthermore, different multiple linear regression models, using uric acid as a categorical variable, were produced where participants with hyperuricemia were assigned “1” and normouricemic participants were assigned “0”. The produced coefficient of this categorical variable, which is a theoretical difference of means between the 2 groups, was presented. In model 1, the multiple linear regression controlled for variation in age and sex. In model 2, the multiple linear regression controlled for variation in age, sex, BMI, smoking and exercise. Although dietary variation may have been a confounder, the Biobank did not provide adequately specific data regarding dietary intake.

Age was categorized into 4 equally sized age groups to help improve model specification: 18-25, 26-29, 30-34, 35-40 years. To control for confounders such as smoking, the questionnaire data collected by QBB was used to categorize participants into 3 groups. Participants were assigned 0 if they have never smoked or ‘only smoked once or twice’; participants were assigned 1 if they are former smokers; participants were assigned 2 if they are current smokers.

To control for exercise, questionnaire data collected by QBB was used to categorize participants into 3 groups. Participants were assigned 0 if they do not exercise; participants were assigned 1 if they carry-out walking exercise only; participants were assigned 2 if they carry-out high-intensity exercise. Post-hoc power calculation was performed using uric acid levels mean among normouricemic (*N*= 687) and hypyeruricemic participants (*N*= 184) and found to be 100% power.

## Results

### Baseline characteristics

This cohort comprises of young Qatari participants, and thus the mean age is 29.4 years and the cohort contains 397 male participants and 474 female participants. The mean serum uric acid level was 293.2 µmol/L (4.93 mg/dL), and the mean BMI was 27.8 kg/m^2^. The baseline characteristics’ means and standard deviations (or medians and interquartile ranges, respectively, for non-normally distributed variables) for the variables are displayed in Table 1.

**Table 1:** Summary of Baseline Characteristics of the Sample

Variable (*N*=871)	N (%) or Mean (SD) or Median (IQR)
Male Female	397 (46%) 474 (54%)
Age	29.43 (5.98)
Serum Uric Acid (µmol/L)	293.2 (80.9)
BMI (Kg/m^2^)	27.85 (6.24)
Waist-to-Hip ratio	1.03 (0.27)
Hemoglobin (g/dL)	13.48 (1.9)
Red blood cells (10^6^/µL)	4.96 (0.58)
Folate (nmol/L)	20.45 (7.02)
TIBC (µmol/L)	61.5 (9.82)
White blood cells (10^3^/µL)	6.79 (1.94)
Monocyte percentage (%)	7.37 (1.87)
Platelets (10^3^/µL)	252.14 (64.93)
*Triglyceride (mmol/L)	0.96 (6.1, 8.5)
HDL-c (mmol/L)	1.41 (0.38)
LDL-c (mmol/L)	2.81 (0.75)
*C-peptide (ng/mL)	1.87 (1.37, 2.73)
Glucose (mmol/L)	5 (0.73)
HbA_1c_ (%)	5.23 (0.42)
*Ferritin (µg/L)	35 (11,92)
*MHR	5.23 (4.02, 6.84)

*Variables are not normally distributed, and accordingly their medians are presented instead of their means, and their interquartile ranges are presented instead of their standard deviations.

### Prevalence of asymptomatic hyperuricemia

We observed that the prevalence of asymptomatic hyperuricemia in 18-40-year-old healthy Qataris, defined as participants with uric acid greater than 356 µmol/L (6.0 mg/dL) was 21.2% (95% CI [18.6%, 24.1%]). We also divided the cohort into two strata based on their age, and we found that the prevalence of asymptomatic hyperuricemia in 18-29-year-old Qataris was 18.2% (95% CI [14.8%, 22.1%], and in 30-40-year-old Qataris was 24.5% (95% CI [20.4%, 28.9%]).

### Associations of asymptomatic hyperuricemia with hematological indices

Table 2 displays the associations between asymptomatic hyperuricemia and hematological indices investigated using t-tests. The data show that the hyperuricemic group had statistically significantly higher levels of hemoglobin, RBC and monocyte percentage, but statistically significantly lower levels of folate and TIBC. Although all the outcomes, except for WBC, display statistically significant differences, none of the differences were considerably large.

**Table 2:** Comparison of Normouricemic and Hyperuricemic Groups

Parameter	Normouricemic (*N*=678) Mean (SD)	Hyperuricemic (*N*=184) Mean (SD)	p-value
Hemoglobin (g/dL)	13.17 (1.88)	14.60 (1.56)	**<0.001**
Folate (nmol/L)	20.83 (7.07)	19.05 (6.66)	**0.002**
RBC (10^6^/µL)	4.87 (0.57)	5.29 (0.51)	**<0.001**
TIBC (µmol/L)	62.44 (10.08)	58.03 (7.89)	**<0.001**
WBC (10^3^/µL)	6.76 (2.00)	6.91 (1.72)	0.348
Monocyte (%)	7.27 (1.84)	7.74 (1.97)	**0.002**
Platelet (10^3^/µL)	255.5 (65.82)	239.7 (60.11)	**0.004**
HDL (mmol/L)	1.47 (0.37)	1.19 (0.29)	**<0.001**
LDL (mmol/L)	2.75 (0.73)	3.07 (0.79)	**<0.001**
*Triglycerides (mmol/L)	0.90 (0.63, 1.30)	1.2 (0.81, 1.60)	**<0.001**
*C-peptide (ng/mL)	1.77 (1.31, 2.55)	2.38 (1.72, 3.49)	**<0.001**
Glucose (mmol/L)	4.94 (0.74)	5.21 (0.63)	**<0.001**
HbA_1c_ (%)	5.21 (0.39)	5.32 (0.50)	**0.002**
*Ferritin (µg/L)	25 (9, 67)	94 (53, 146.5)	**<0.001**
*MHR	4.94 (3.82, 6.46)	6.60 (5.18, 8.08)	**<0.001**

*Variables are not normally distributed, and accordingly their medians are presented instead of their means, and their interquartile ranges are presented instead of their standard deviations, and Mann-Whitney U tests were used to produce their p-values instead of t-tests.

The correlation coefficients in Table 3 display similar statistically significant changes where all the outcomes except WBC, show statistically significant associations with serum uric acid. Whilst these are statistically significant associations, only hemoglobin and RBC count display impressive degrees correlations, with R^2^ values exceeding 10%.

**Table 3:** Correlation Coefficient with serum uric acid levels

Parameter	Correlation coefficient	R^2^	p-value
Hemoglobin (g/dL)	0.482	0.232	**<0.001**
Folate (nmol/L)	-0.122	0.015	**<0.001**
RBC (10^6^/µL)	0.434	0.188	**<0.001**
TIBC (µmol/L)	-0.288	0.083	**<0.001**
WBC (10^3^/µL)	0.029	0.001	0.387
Monocyte (%)	0.139	0.019	**<0.001**
Platelets (10^3^/µL)	-0.171	0.029	**<0.001**
HDL (mmol/L)	-0.429	0.184	**<0.001**
LDL (mmol/L)	0.221	0.049	**<0.001**
*Triglycerides (mmol/L)	0.308	NA	**<0.001**
*C-peptide (ng/mL)	0.274	NA	**<0.001**
Glucose (mmol/L)	0.207	0.043	**<0.001**
HbA_1c_ (%)	0.147	0.022	**<0.001**
*Ferritin (µg/L)	0.568	NA	**<0.001**
*MHR	0.409	NA	**<0.001**

*Variables are not normally distributed, and thus Spearman’s rank correlation coefficient was used.

**Fig. 1:**
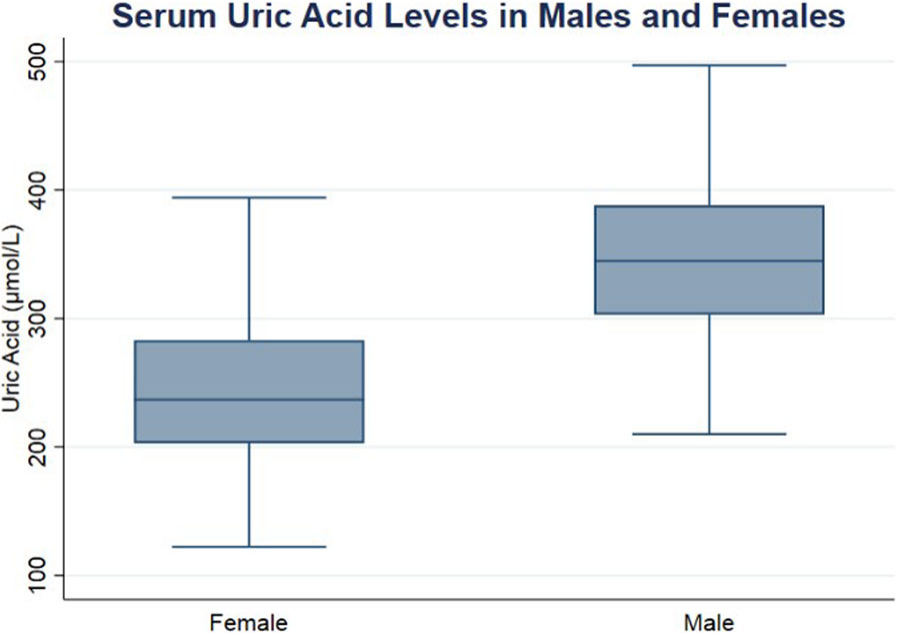
Box plot of gender-based serum uric acid levels (µmol/L). (Females *N*= 474, Males *N*= 397)

Since serum uric acid levels are different between males and females (Fig. 1), data were further examined and adjusted for confounders. Using multiple linear regression analysis, model 1 was adjusted for age groups and gender and model 2 was adjusted for age groups, gender, BMI, smoking and exercise. No statistically nor clinically significant associations were observed (Table 4) which is in contrast to the significant associations observed between uric acid and hematological indices in the correlation tests of Table 3.

**Table 4:** Multiple Linear Regression Models^#^ predicting the difference of means for each variable of interest between hyperuricemic and normouricemic groups

	^$^Model 1				^$^Model 2			
Parameter	**Coefficient**	**95% CI**	**p-value**	**R**^**2**^	**Coefficient**	**95% CI**	**p-value**	**R**^**2**^
Hemoglobin (g/dL)	-0.12	(-0.35, 0.11)	0.315	0.56	-0.14	(-0.39, 0.10)	0.259	0.57
Folate (nmol/L)	-0.55	(-1.81, 0.70)	0.389	0.03	-0.37	(-1.67, 0.93)	0.576	0.04
RBC (10^6^/µL)	0.04	(-0.04, 0.12)	0.362	0.37	-0.01	(-0.10, 0.08)	0.785	0.35
TIBC (µmol/L)	0.07	(-1.54, 1.68)	0.934	0.18	0.00	(-1.72, 1.72)	0.999	0.19
WBC (10^3^/µL)	0.16	(-0.20, 0.51)	0.386	0.00	-0.12	(-0.50, 0.26)	0.534	0.04
Monocytes (%)	-0.01	(-0.34, 0.32)	0.960	0.06	0.19	(-0.17, 0.54)	0.298	0.06
Platelet (10^3^/µL)	8.82	(-2.32, 19.96)	0.121	0.11	-0.38	(-12.30, 11.54)	0.950	0.13
HDL (mmol/L)	-0.14	(-0.20, -0.08)	**<0.001**	0.21	-0.07	(-0.14, -0.01)	**0.019**	0.26
LDL (mmol/L)	0.18	(0.05, 0.31)	**0.008**	0.11	0.13	(-0.01, 0.27)	0.065	0.11
Triglycerides (mmol/L)	0.20	(0.09, 0.31)	**0.001**	0.13	0.12	(-0.01, 0.25)	0.066	0.15
C-peptide (ng/mL)	0.75	(0.46, 1.04)	**<0.001**	0.05	0.38	(0.06, 0.69)	**0.018**	0.12
Glucose (mmol/L)	0.15	(0.02, 0.28)	**0.021**	0.05	0.06	(-0.08, 0.21)	0.395	0.07
HbA_1c_ (%)	0.10	(0.02, 0.17)	**0.011**	0.04	0.04	(-0.04, 0.12)	0.358	0.07
*Ferritin (Males) (µg/L)	16.01	(1.93, 30.09)	**0.026**	0.04	8.08	(-7.18, 23.34)	0.298	0.07
*Ferritin (Females) (µg/L)	15.06	(0.19, 29.93)	**0.047**	0.01	13.72	(-0.27, 27.7)	0.054	0.00
MHR	0.62	(0.25, 1.00)	**0.001**	0.21	0.47	(0.06, 0.89)	**0.026**	0.21

^#^The linear regression models were constructed using categorical uric acid groups where normouricemics were labeled 0 and hyperuricemics were labeled 1, and thus the coefficients displayed indicate the mean difference in the outcome variables between the hyperuricemic and normouricemic groups.

^$^Model 1 is adjusted for age groups and sex. Model 2 is adjusted for age groups, sex, BMI, smoking and exercise. Adjusted R2 values have been displayed.

*The regression models pertaining to ferritin were stratified by sex to avoid model misspecification.

### Associations of asymptomatic hyperuricemia with lipid profile

We observed that hyperuricemic group had statistically significantly lower HDL-c levels but statistically significantly higher LDL-c and triglyceride levels (Table 2). The hyperuricemic group’s lower mean HDL-c (0.28 mmol/L lower than the control group) is significant when considering that HDL-c is a prominent antioxidant. The correlation tests shown in Table 3 also revealed the statistically significant negative association between uric acid and HDL-c levels. Furthermore, statistically significant positive associations between uric acid, and triglyceride and LDL-c levels were observed. The associations are evident by the large correlation coefficients, especially in the case HDL-c.

Using multiple linear regression models (Table 4), all three lipids coefficients were statistically significant in model 1, which controls for age and gender variation, indicating that HDL-c is statistically significantly lower, while LDL-c and triglycerides are statistically significantly higher, in the hyperuricemic group compared to the control group. When adjusting for age, gender, BMI, exercise and smoking in model 2, statistical significance was attributable to HDL-c only, which is significantly lower in the hypeuricemic group compared to control group.

### Associations of asymptomatic hyperuricemia with glycemic indices

The hyperuricemic group has statistically significantly higher levels of all glycemic indices (Table 2). Correlation tests revealed modest but statistically significant positive associations between uric acid levels and all 3 glycemic indices. In Model 1 of the multiple linear regression (Table 4), the coefficients of all three glycemic indices were statistically significant, but their magnitudes were again only modest. Upon controlling for additional confounders in Model 2, only the coefficient of c-peptide was statistically significant, while HbA_1c_ and random glucose lost their statistical significance. Notably, HbA_1c_ was not statistically significantly different between the normouricemic and hyperuricemic groups in Model 2 of Table 4. However, HbA_1c_ maintained the statistically significant association with uric acid in Model 2 when using uric acid on a continuous scale in Appendix 1.

### Associations of asymptomatic hyperuricemia with subclinical inflammation markers

**Fig. 2:**
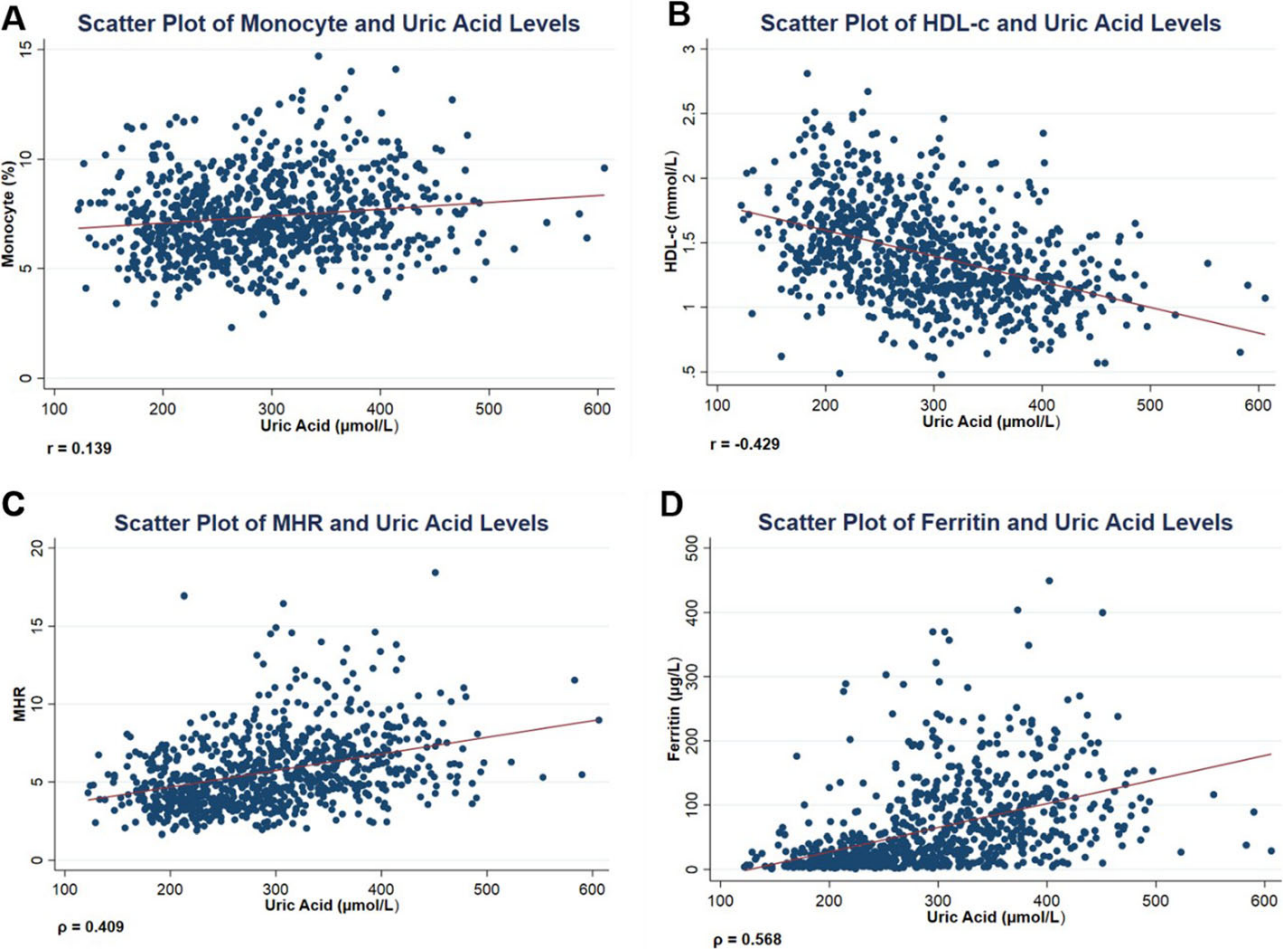
Scatter plots depicting uric acid association with subclinical inflammation markers. **A**: monocyte %; **B**: HDL-c; **C**: MHR; **D**: Ferritin

Ferritin and MHR are sensitive markers of subclinical inflammation, and their levels are notably higher in the hyperuricemic group (Table 2). As presented in scatter plots (Fig. 2), correlation between uric acid and inflammatory markers exhibits remarkable effect size and statistical significance (Table 3), as evident by the large correlation coefficients regarding ferritin (ρ = 0.565) and MHR (ρ = 0.409). However, when multiple linear regression analysis was used (Table 4), ferritin succumbed to severe model misspecification. Therefore, gender stratification was implemented to improve the model specification adequately.

The coefficient of MHR was statistically significant and considerably large in both models (Table 4). Moreover, the coefficients of ferritin in males and females were statistically significant in Model 1. When adjusting for BMI, smoking and exercise in Model 2, ferritin coefficient was still statistically significant in females but not in males.

## Discussion

Here we report the prevalence of asymptomatic hyperuricemia among young healthy Qatari adults is 21.2%. We have identified varying degrees of association between elevated uric acid levels and markers of dyslipidemia, prediabetes, and subclinical inflammation. Our findings particularly support a statistically and clinically significant association between serum uric acid levels and markers of subclinical inflammation, ferritin and MHR, as well as HDL-c levels.

The prevalence of asymptomatic hyperuricemia in our Qatari cohort is 21.2% (95% CI [18.6, 24.1]), which is slightly higher than the estimated global prevalence range of 10-20% (11) but considerably higher than that reported in regional studies. Al-Arfaj conducted a study in Saudi Arabia that included 487 participants, and found that the prevalence of hyperuricemia was 8.42% [24]. However, there are many reasons for the large discrepancy between the prevalence of hyperuricemia we report in Qatar and that reported in Saudi Arabia. Firstly, Al-Afraj study cohort included participants within the age 14-83 years, while our cohort was limited to young adults in the range of 18-40 years. Secondly, we determined asymptomatic hyperuricemia at a lower of 356 µmol/L indiscriminate of gender; however, Al-Arfaj’s study used a higher threshold for males at 420 µmol/L (7.0 mg/dL), and 356 µmol/L (6.0 mg/dL) for females. The higher threshold used may explain the lower prevalence of hyperuricemia. Lastly, Al-Arfaj’s study was conducted between 1998 and 1999, and considering the recent global obesity epidemic, it is reasonable to speculate that the prevalence of asymptomatic hyperuricemia may be currently higher.

The gender effect on uric acid levels is observed in our study. The difference in serum uric acid levels between male and female populations are shown in (Fig. 1). A study by Zheng et al. investigated the gender differences in the association of serum uric acid and cardiovascular disease risk, and it explored the differences in serum uric acid levels. They showed that lower levels of uric acid in females may be related to higher levels of estrogen in women, as estrogen was previously associated with increase in uric acid excretion by the kidneys [25]. These findings could provide an explanation to the lower levels of serum uric acid found in females as opposed to males in our cohort.

In this report, we initially found significant associations between uric acid and hematological indices in the t-tests and correlations. However, after adjusting for confounders using multiple linear regression analysis, no significant associations were found. This suggests that the significant associations previously seen in the t-tests and correlations were confounded by age and gender variation, and uric acid has no causal relationship with the hematological indicators. Moreover, a significant association between asymptomatic hyperuricemia and lipid profile was identified in our study, even after adjusting for confounders such as age, sex, exercise, smoking and BMI. Like Model 1, the magnitudes of the coefficients in Model 2 are all modest and clinically significant. Several previous studies reported the positive associations of uric acid with triglycerides and LDL-c and the inverse association with HDL-c levels [26–28], which lend support to our findings. Study by Lima et al. [29] evaluated the lipid metabolism modulating properties of uric acid in different tissues [28]. In their study, they reviewed clinical and epidemiological papers that suggest hyperuricemia may lead to metabolic alterations, resulting in postprandial increases in triglyceride levels, accumulation of triglycerides in hepatic tissue, as well as disturbances in the insulin response in liver, adipose and muscle tissues. This may provide a rationale for the association between asymptomatic hyperuricemia and lipid profile indicators found in our study. The significant inverse correlation between asymptomatic hyperuricemia and HDL-c suggests that hyperuricemia may be associated with disturbances in lipid homeostasis, and thus uric acid levels may be used as a biomarker to predict the future incidence of dyslipidemia in a currently healthy individual.

In our study, we found statistically significant positive associations between asymptomatic hyperuricemia and markers of diabetes, such as random glucose, c-peptide and HbA_1c_ levels using t-tests and correlation tests. However, after adjusting for confounders such as age, sex, BMI, smoking and exercise, this association only remains significant for c-peptide, a biomarker of insulin production. Although the coefficient of c-peptide is not clinically significant, it is the only marker that maintained its statistical significance upon controlling for all the identified confounders (Table 4, Model 2). This is noteworthy because it is consistent with our findings (Table 3) which showed that c-peptide had the highest correlation coefficient of the glycemic markers. As previously discussed, we have identified that increasing uric acid levels may be associated with increased insulin production, evidenced by the statistically significant association uric acid has with c-peptide levels. However, this finding may possibly be contradictory with previous studies, which suggest that uric acid may hinder the production of insulin via inhibiting the proliferation of beta-cells in the pancreas, as well as decreasing gene expression of insulin [30, 31]. Therefore, the positive association between glucose and asymptomatic hyperuricemia may be scientifically plausible since several studies have stated that uric acid levels may contribute to elevated serum glucose by increasing hepatic gluconeogenesis and inhibiting insulin-mediated entry of glucose into cells [10, 32].

Both random glucose and c-peptide, although within their normal ranges, could be indicators for the risk of progression to prediabetes. Slightly elevated random glucose and c-peptide levels might suggest an underlying disruption of glucose homeostasis, which might later lead to the pathogenesis of prediabetes and progression to diabetes. A study on a large Qatari cohort found that c-peptide could be used as a biomarker to predict diabetes [33]. The same study also highlighted the association between uric acid and diabetes, which reiterates the findings of our report, especially since the cohorts of both studies were in Qatar.

Herein this study, strong positive associations between asymptomatic hyperuricemia and markers of subclinical inflammation, before and after adjustment for confounders were observed. Study by Joosten et al. [34] extensively reviewed the pro-inflammatory properties of uric acid, both in crystal and soluble forms [34]. Soluble uric acid contributes to oxidative stress intracellularly by promoting formation of reactive oxygen species via activation of NADPH oxidase. This enzyme attaches to the mitochondrial membrane resulting in mitochondrial oxidative stress. Oxidative stress on a cellular level triggers pro-inflammatory signaling and activates the innate immune system. This sequence of events may explain how uric acid triggers subclinical inflammation in non-crystal forming hyperuricemia (15). Furthermore, the significant association between asymptomatic hyperuricemia and markers of subclinical inflammation, in otherwise healthy participants, suggests that uric acid might play a role in the pathogenesis of chronic inflammatory conditions, such as diabetes, cardiovascular diseases and dyslipidemia. The associations we report between uric acid, and markers of prediabetes and dyslipidemia, further support this notion. Several other papers also reported finding similar associations between uric acid and such conditions [35–37]. Thus, the potential to use uric acid as a biomarker to predict the progression to chronic inflammatory disorders needs to be further investigated, as uric acid is already suggested to be a pro-inflammatory molecule that possibly mediates subclinical inflammation.

A unique aspect of our study is the use of a cohort of young adults (18-40 years) who are devoid of any comorbidities. Exploring this age group might provide important insight about the potential risk that a young adult may later develop chronic disease conditions. Our report is also distinct in that it is the first study carried out to evaluate the prevalence of asymptomatic hyperuricemia, as well as its potential use as a biomarker for disorders of metabolic syndrome, in a Qatari cohort. The limitations of this study include the following: due to the cross-sectional nature of our study design, any assumptions on directionality regarding the causality of uric acid on the metabolic disorders would be void and invalid. Given the current study design, it is difficult to determine whether hyperuricemia leads to chronic inflammatory conditions, or these conditions elevate uric acid levels. Therefore, cohort studies are needed to ascertain the directionality of these associations. Another limitation concerns predictions of the significance of the relationship between uric acid and glycemic indices. Fasting blood glucose is a fundamental component when assessing a person’s glycemic status; however, the data on the fasting blood glucose levels in this cohort are not available. Lastly, although we adjusted for numerous possible confounders in multiple linear regression analysis, we were unable to control for dietary variation, which is an important possible confounder. A considerable limitation is the lack of diet data for this cohort specifically on the consumption of meat, fructose or alcohol, all of which affect serum uric acid levels [38], thus may have been possible confounders. Moreover, evidence on confounders in the literature is often conflicting, which made it difficult to determine possible confounders. Nevertheless, we selected confounders very conservatively to prevent inducing bias such as collider-stratification bias or over-adjustment bias.

## Conclusions

The prevalence of asymptomatic hyperuricemia in young Qatari adult population was found to be 21.2%. Considering this high prevalence rate, asymptomatic hyperuricemia has implications for the general Qatari population, as our findings indicate that asymptomatic hyperuricemia is associated with markers of dyslipidemia, prediabetes, and subclinical inflammation. Therefore, our study would advocate for further clinical studies to be carried out investigating the nature of the association between asymptomatic hyperuricemia and subclinical metabolic disturbances. Finally, in light of our study’s findings, we would recommend that subjects with asymptomatic hyperuricemia are informed about the potential risk of progression to chronic diseases and are recommended suitable lifestyle modifications to prevent the incidence of such disorders.

## Data Availability

The datasets analyzed during the current study are obtained under Material Transfer agreement from Qatar Biobank repository in Doha, Qatar [https://www.qatarbiobank.org.qa/], and so are not publicly available. However, if access to the private data upon reasonable request is needed, Dr Susu Zughaier (szughaier@qu.edu.qa) will facilitate communication with Qatar Biobank.
